# With Chitosan and PLGA as the Delivery Vehicle, *Toxoplasma gondii* Oxidoreductase-Based DNA Vaccines Decrease Parasite Burdens in Mice

**DOI:** 10.3389/fimmu.2021.726615

**Published:** 2021-08-27

**Authors:** Zhengqing Yu, Wandi Cao, Xuchen Gao, Muhammad Tahir Aleem, Junlong Liu, Jianxun Luo, Ruofeng Yan, Lixin Xu, Xiaokai Song, Xiangrui Li

**Affiliations:** ^1^Ministry of Education (MOE) Joint International Research Laboratory of Animal Health and Food Safety, College of Veterinary Medicine, Nanjing Agricultural University, Nanjing, China; ^2^State Key Laboratory of Veterinary Etiological Biology, Key Laboratory of Veterinary Parasitology of Gansu Province, Lanzhou Veterinary Research Institute, Chinese Academy of Agricultural Sciences, Lanzhou, China

**Keywords:** *Toxoplasma gondii*, oxidoreductase, chitosan, PLGA, immune protection, mouse

## Abstract

*Toxoplasma gondii* (*T. gondii*) is an intracellular parasitic protozoan that can cause serious public health problems. However, there is no effectively preventive or therapeutic strategy available for human and animals. In the present study, we developed a DNA vaccine encoding *T. gondii* oxidoreductase from short-chain dehydrogenase/reductase family (TgSDRO-pVAX1) and then entrapped in chitosan and poly lactic-co-glycolic acid (PLGA) to improve the efficacy. When encapsulated in chitosan (TgSDRO-pVAX1/CS nanospheres) and PLGA (TgSDRO-pVAX1/PLGA nanospheres), adequate plasmids were loaded and released stably. Before animal immunizations, the DNA vaccine was transfected into HEK 293-T cells and examined by western blotting and laser confocal microscopy. Th1/Th2 cellular and humoral immunity was induced in immunized mice, accompanied by modulated secretion of antibodies and cytokines, promoted the maturation and MHC expression of dendritic cells, and enhanced the percentages of CD4^+^ and CD8^+^ T lymphocytes. Immunization with TgSDRO-pVAX1/CS and TgSDRO-pVAX1/PLGA nanospheres conferred significant immunity with lower parasite burden in the mice model of acute toxoplasmosis. Furthermore, our results also lent credit to the idea that TgSDRO-pVAX1/CS and TgSDRO-pVAX1/PLGA nanospheres are substitutes for each other. In general, the current study proposed that TgSDRO-pVAX1 with chitosan or PLGA as the delivery vehicle is a promising vaccine candidate against acute toxoplasmosis.

## Introduction

As an obligate intracellular parasite, *Toxoplasma gondii* (*T. gondii*) can infect almost all warm-blooded vertebrates and are widespread in humans (1, 2). Most *T. gondii* infections are asymptomatic, but a serious threat can be posed to immunocompromised patients and those with congenital toxoplasmosis (3). Based on the published paper, toxoplasmosis may occur by ingestion of contaminated meat, water, or even vegetables (4–6). Such high disease burden makes *T. gondii* one of the most important food-borne microorganisms and emphasizes the necessity to intervene (7). Invalid to the bradyzoites of *T. gondii*, pyrimethamine (PYR) and sulfadiazine (SDZ) have been permitted for the treatment of tachyzoites (8), implying that chronic toxoplasmosis caused by bradyzoites cannot be eradicated (9). Thus, development of effective protective and therapeutic strategies needs to be improved (10). Currently, effective treatments for toxoplasmosis are still unavailable, and the prevention strategies are considered effectively (11). Commercially available Ovilis Toxovax® (Intervet Inc., Angers, France), a live attenuated vaccine, prepared by the *T. gondii* S48 strain has been licensed in the prevention of toxoplasmosis in goats and sheep (12). However, the Food Standards Agency questioned its protective immunity in tissue cyst formation (13). In general, it is an urgent and valued requirement to develop an effective vaccine against *T. gondii* infection.

A safe and effective vaccine against *T. gondii* may be the promising way to protect against toxoplasmosis (14). Currently, studies on vaccines against *T. gondii* have mainly focused on finding promising vaccine targets and demonstrating its protections in immunity (15). Eliciting strong cellular and humoral immunity against *T. gondii*, an effective vaccine target should be highly immunogenic and improbable to induce autoimmune and allergic reactions (15). Numerous vaccine targets have been evaluated in different types of vaccines against *T. gondii*, and these targets mainly include dense granule proteins (16), surface proteins (17), rhoptry proteins (18), microneme proteins (19), etc. However, these vaccine targets cannot fully protect against the virulent challenge, and a suitable vaccine is still unavailable (20–22). The short-chain dehydrogenases/reductases family (SDR family) is a large family of enzymes in eukaryotes and a potential vaccine target (23), which is involved in the synthesis of molecules, including lipids, vitamins, drugs, and carbohydrates (24). A 368-amino acid protein encoded as “oxidoreductase” from the *T. gondii* SDR family in the Uniprot database (https://www.uniprot.org/) aroused our particular attention. The NADPH-dependent oxidoreductase is characterized by 3-oxoacyl-ACP reductase (NADPH) activity (25, 26) and plays essential roles in fatty acid biosynthesis, and is a critical enzyme in the synthesis of poly(β-Hydroxybutyric acid), β-ketothiolase, and polyhydroxyalkanoate (PHA) synthase (27). It has been demonstrated that the apical complex exerts great functions on the invasion of apicomplexan parasites (28). Harboring metabolic processes that are important in fatty acid biosynthesis, the apical complex is indispensable for parasite viability (29, 30). Thus, construction of specifically targeting the activity of oxidoreductase vaccines looks rational in the prevention of *T. gondii* infection.

With the emergence of new antigens, adjuvants, and therapeutics, vaccines against toxoplasmosis evolved noticeably. In recent years, many studies have evaluated the immune protection triggered by DNA vaccines encoding a single or multicomponent antigen against toxoplasmosis in a mice model (17, 18, 31). As a DNA vaccine vector, pVAX1 is approved by the Food and Drug Administration (FDA). Furthermore, gene expression levels tend to be a key factor to evaluate the protective efficacy of a genetic vaccine. Thus, a delivery system for DNA vaccines should be applied with the aim to enhance the expression of transferred antigens to evoke adequate immune protection (32). To decrease the dose of plasmids and protect the plasmids from degradation, numerous studies have been focused on an efficient delivery system (33). Nowadays, many synthetic delivery systems have been reported for their improvement in immune response (34). Widely used in polymer materials for vaccine and drug delivery system, poly lactic-co-glycolic acid (PLGA) approved by FDA is characterized by easy biodegradability, nontoxicity, and good biocompatibility (35). Also approved for wound dressings by FDA (36), chitosan has been illustrated to be safe in parts of dietary applications (37). Chitosan is also suitable for biomedical applications largely due to being biocompatible, biodegradable, and relatively safe (38–40).

In the present study, we made a new attempt in combining *T. gondii* oxidoreductase from SDR family (TgSDRO) with PLGA and chitosan nanospheres to develop a DNA vaccine against *T. gondii* infection in mice. In addition, the immune protections of two types of nanospheres were also evaluated. In this context, the aim of this study was to develop a DNA vaccine against acute toxoplasmosis delivered by PLGA or chitosan.

## Materials and Methods

### Ethics Statements

All animal experiments were approved by the Animal Ethics committee of responsible authority from the College of Veterinary Medicine, Nanjing Agricultural University, China (permission: NJAU. No20210315014).

### Animals, Cell Lines, and Parasites

Specific pathogen-free (SPF) BALB/c mice (18–22 g, female) and Sprague–Dawley (SD) rats (200–220 g, female) were purchased from Model Animal Research Center of Nanjing University, Nanjing University, China. All animals were strictly housed in a specific pathogen-free environment following the requirements of the Animal Ethics Procedures and Guidelines of the People’s Republic of China.

Human embryonic kidney 293-T (HEK 293-T) cells were obtained from the Institute of Cell Biology, Chinese Academy Sciences, Shanghai, China, and preserved in our laboratory. HEK 293-T cells were cultured in Dulbecco’s Modified Eagle’s Medium (DMEM, Gibco, Carlsbad, CA, USA) containing 10% fetal bovine serum (FBS, Gibco, Carlsbad, CA, USA) and 1% double antibiotics (Carlsbad, Gibco, CA, USA) at 37°C in a 5% CO_2_ atmosphere.

Tachyzoites of the violent *T. gondii* RH strain (type I) were maintained in the Ministry of Education (MOE) Joint International Research Laboratory of Animal Health and Food Safety, College of Veterinary Medicine, Nanjing Agricultural University, Nanjing, China. Tachyzoites used in this study were replicated in BALB/c mice according to a previous study (41).

### Construction of the Eukaryotic Expression Plasmid

Strictly following the instructions, the total RNA of 10^6^ tachyzoites of *T. gondii* was isolated by using the Total RNA Extraction kit (OMEGA Bio-tek, Norcross, GA, USA). Reverse transcription PCR (RT-PCR) was immediately conducted to synthesize the cDNA by a reverse transcription kit (Takara Biotechnology, Dalian, China). Primers amplifying the complete open reading frame (ORF) of TgSDRO were designed based on the conserved domain sequences (CDS) of the TgSDRO gene (GenBank: AAYL02000041). Along with the restriction endonuclease sites (*Eco*RI and *Xho*I) and a Kozak translation initiation sequence, the forward and reverse primers, 5’-CCG GAATTC GCCACC ATGGATATTCAAGCGGTTGGGA-3’ and 5’-CCGCTCGAGTCAGGTGGAATGCGGGGG-3’, were synthesized by the company (Tsingke Biological Technology, Nanjing, China). For PCR amplification, 1.25 U *Ex Taq* DNA polymerase (Takara Biotechnology, Dalian, China), 5 µl 10 × *Ex Taq* buffer, 4 µl dNTP mixtures (Mg^2+^ plus), 20 pmol of each primer, 2 ng cDNA template, and ddH_2_O to make a final volume of 50 µl were involved in each reaction. The amplification was determined by an amplifier (Thermo Scientific, Waltham, MA, USA) using the following program: preheating (5 min at 95°C), amplification for 35 cycles of 30 s at 95°C, 30 s at 64°C and 70 s at 72°C, and the final extension (5 min at 72°C). Then, 10 μl PCR amplicons were analyzed in a 1.0% agarose gel containing 0.01% ethidium bromide. The single band with expected base pairs was then purified by using a Gel Eradication Kit (OMEGA Bio-tek, Norcross, GA, USA). After being digested with *Eco*RI and *Xho*I restriction endonuclease (Takara Biotechnology, Dalian, China), the obtained PCR amplicons were inserted into the linear pVAX1 vector (Invitrogen Biotechnology, Shanghai, China) by using T4 DNA ligase (Takara Biotechnology, Dalian, China). The recombinant plasmids were identified by double restriction enzyme digestion and then sequenced by the ABI PRISM™ 3730 XL DNA Analyzer (Applied Biosystems, Waltham, MA, USA). The positive plasmid was transferred to *Escherichia coli* (*E. coli*) DH5α (Invitrogen Biotechnology, Shanghai, China).

To obtain a large quantity of recombinant plasmid, *E. coli* DH5α carrying the recombinant plasmid was amplified in a Luria Bertani (LB) medium containing 100 μg/ml kanamycin monosulfate until the OD600 reached 0.6 (37°C and shaking at 180 rpm). Endo-free DNA plasmid was extracted by a commercially available kit (Vazyme, Nanjing, China) in a large quantity, and then the level of endotoxin was determined by a ToxinSensor™ Chromogenic LAL Endotoxin Assay Kit (GeneScript, Piscataway, NJ, USA). After concentration determination by a nanodrop microvolume spectrophotometer (Thermo Scientific, Waltham, MA, USA), the recombinant plasmids were stored at -20°C until use.

### Recombinant Plasmids Transfection *In Vitro*

The liposomal transfection method was used for the delivery of plasmids in HEK 293-T cells (1 × 10^6^ cells) cultured in a 6-well plate (Costar, Cambridge, MA, USA) with 70–90% confluence, then a Lipofectmine™ 3000 reagent (Invitrogen Biotechnology, Shanghai, China) was used as the transfection reagent for making the plasmid–lipid complex according to the instructions. The transfected cells were incubated for 15 min before replacing the medium with 2 ml of fresh medium. Three days later, the cultured medium was removed, and the cells were washed with phosphate buffered saline (PBS) for three times. Then cells were scraped into 250 μl RIPA lysis buffer (Beyotime, Shanghai, China) containing 10 μl protease and phosphatase inhibitor (Beyotime, Shanghai, China). Tip sonication (Scientz Biotechnology, Ningbo, China) was conducted in a continuous mode for 2 s at 2 s (5 min in total) under the output power of 20 W, and then the cell lysates were stored at -20°C until use.

To obtain the cell culture medium containing recombinant protein, the culture medium was first renewed after a 3-day cultivation, and then the transfected cells were scraped into the medium; then the tip sonication was conducted in the same continuous mode mentioned above under sterile condition. The cell culture medium was collected and stored at -20°C after centrifugation at 12,000 rpm for 5 min at 4°C.

### Immunofluorescence Staining

To obtain the serum against *T. gondii*, two SD rats were challenged with 10^3^ tachyzoites of *T. gondii* RH strain intraperitoneally. One month later, the serum containing the antibodies against *T. gondii* was harvested. All sera were kept at -20°C until use.

The transfected HEK 293-T cells were prepared following the method described in the section *Recombinant Plasmids Transfection in Vitro*. With 3-day stationary cultivation after transfection in the medium, cells were washed with PBS for three times and fixed with 1 ml 4% paraformaldehyde for 12 h at 4°C. Permeabilized with tris buffered saline (TBS) containing 0.1% Triton X-100 (TBSx) for 3 min on a rotary shaker at 50 rpm, cells were subsequently incubated with TBSx containing 5% BSA and 1% serum obtained from *T. gondii*-infected rats for 1 h on a rotary shaker at 37°C. Diluted 1:500 in TBSx containing 5% BSA, CY3-conjugated anti-rat IgG (Sigma, Saint Louis, MO, USA) was added to the cells before washing three times in TBSx. After being incubated for 1 h at 37°C on a rotary shaker, 500 μl of 4’,6-diamidino-2-phenylindole (DAPI) staining solution (Beyotime, Shanghai, China) was added in each well. After being stained for 5 min at 37°C on a rotary shaker, DAPI staining solution was removed, and the cells were washed three times in TBSx. Cover lips were mounted with 300 μl anti-fade mounting medium (Beyotime, Shanghai, China) and the image was taken by a Nikon A1 plus laser scanning confocal microscopy (Nikon Corporation, Tokyo Metropolis, Japan).

### Western Blot Analysis

HEK 293-T cell lysates were added with 10 × loading buffer and heated at 95–100°C for 5 min. Separated by 12% SDS-PAGE, HEK 293-T cell lysates were electro-transferred onto polyvinylidene difluoride (PVDF) membranes (Millipore, Billerica, MA, USA). The membranes were then incubated with 5% (*w/v*) BSA in TBS containing 0.5% (*v/v*) tween 20 (TBST) for 2 h at 37°C on a rotary shaker with 50 rpm. After three times washing in TBST, the membranes were subsequently incubated with TBST containing 1% (*v/v*) rat serum at 4°C overnight with constant shaking. Washed with TBST in triplicate, the membranes were incubated with HRP-conjugated anti-rat IgG (1:5,000, eBioscience, San Diego, CA, USA) dissolved in TBST for 1 h at 37°C on a rotary shaker. After three times washing in TBST, the membranes were incubated with newly prepared 3,3’-diaminobenzidine (DAB, Sigma-Aldrich, Saint Louis, MO, USA) for 10 min.

### Synthesis of Vaccines Delivered by PLGA

According to a previous study (42), the double emulsion solvent evaporation technique (*w/o/w*) was used for the synthesis of PLGA nanospheres loaded with TgSDRO-pVAX1 plasmid (TgSDRO-pVAX1/PLGA nanospheres) with minor modifications. Briefly, to construct the organic phase, 5 mg of PLGA (MW: 40,000–75,000 Da, LA/GA: 65/35, Sigma, Saint Louis, MO, USA) was dissolved in 1 ml dichloromethane (DCM, Sigma, Saint Louis, MO, USA). Subsequently, 2 mg (the concentration was 1 mg/ml) purified plasmids were dropwise dissolved in 2 ml of 6% (*w/v*) polyvinyl alcohol (PVA, MW: 31,000–75,000 Da, Sigma, Saint Louis, MO, USA) with a plastic pipette tip at room temperature to construct the aqueous phase. For the 6% PVA solution, PVA was dissolved in double-distilled water and passed through a 0.22-μm filtering membrane (Millipore, Billerica, MA, USA). After the aqueous phase was thoroughly mixed on a vortex mixer for 2 min, the organic phase was dropped into the aqueous phase on a magnetic stirrer (400 rpm) using a plastic pipette tip. To form the *w/o/w* emulsions, tip sonication (Scientz Biotechnology, Ningbo, China) was then conducted in a continuous mode for 5 s at 5 s (4 min in total) under the output power of 50 W in an ice bath. To evaporate DCM, the *w/o/w* emulsions were stirred at 400 rpm on a magnetic stirrer at 4°C overnight. After being centrifuged at 35,000 rpm for 25 min at 4°C, the nanospheres were collected and dissolved in double-distilled water, and the supernatant was also obtained to calculate the total amount of plasmids. The obtained nanospheres were passed through a 0.22-μm filtering membrane, then frozen at -80°C for at least 2 h. The frozen PLGA nanospheres were quickly transferred to a vacuum freeze dryer (Labconco, Kansas City, MO, USA) until completely freeze-dried. The empty PLGA nanospheres were also prepared by replacing the TgSDRO-pVAX1 plasmid with PBS, and the synthesized PLGA nanospheres were stored at 4°C until use.

### Synthesis of Vaccines Delivered by Chitosan

The ionic gelation technique was conducted for the synthesis of chitosan nanospheres loaded with TgSDRO-pVAX1 plasmid (TgSDRO-pVAX1/CS nanospheres) according to a previous study (43). Briefly, 0.1 g of chitosan (MW 50–190 kDa, Sigma, Saint Louis, MO, USA) was dissolved in 50 ml of 1% (*v/v*) aqueous solution of acetic acid. The chitosan solution was then thoroughly mixed on a magnetic stirrer to make chitosan fully dissolved, then the pH value was adjusted by 2 M NaOH solution. Sodium tripolyphosphate (0.02 g; TPP, Aladdin, Shanghai, China) was dissolved in 10 ml double-distilled water and passed through a 0.22-μm filtering membrane (Millipore, Billerica, MA, USA). TPP solution (4 ml) was dropwise added to 20 ml of chitosan solution on a magnetic stirrer at a bath temperature of 30°C, and then 4 mg (the concentration was 1 mg/ml) purified plasmids were dropwise added in. Constant stirring continued at least for 20 min to make chitosan fully dissolved. Then tip sonication was performed in a continuous mode for 5 s at 5 s (2 min in total) under an output power of 50 W in an ice bath. After being centrifuged at 40,000 rpm for 25 min at 4°C, the nanospheres were collected and dissolved in double-distilled water, and the supernatant was also collected to calculate the total amount of plasmids. Before freezing at -80°C for at least 2 h, the nanospheres were passed through a 0.22-μm filtering membrane. The frozen chitosan nanospheres were then transferred to a vacuum freeze dryer until completely freeze-dried. To synthesize empty chitosan nanospheres, PBS, instead of TgSDRO-pVAX1 plasmid, was conducted. The synthesized chitosan nanospheres were stored at 4°C until use.

### Nanospheres Characterization

To investigate the encapsulation efficiency (EE) and the loading capacity (LC), the concentration of free plasmids in the supernatant was quantified by a nanodrop microvolume spectrophotometer, and the total amount of free plasmids in the supernatant could be obtained. The EE and LC can be calculated by formula (1) and formula (2), respectively.

1 $$\text{EE}(\%)=\frac{\text{Total plasmid-Free plasmid}}{\text{Total plasmid}}\times 100\%$$

2 $$\text{LC}(\%)=\frac{\text{Total plasmid-Free plasmid}}{\text{Weight of nanospheres}}\times 100\%$$

To characterize the features of TgSDRO-pVAX1/PLGA and TgSDRO-pVAX1/CS nanospheres, the nanospheres were sent to Nanjing Agriculture University for scanning electron microscope (SEM) observation by using Hitachi SU8010 (Tokyo, Japan). By using the ImageJ software version 1.8 (NIH Image, Bethesda, MD, USA), the average diameter of nanospheres was determined by measuring five arbitrary nanospheres.

To investigate the integrity of prepared nanospheres, synthesized PLGA and chitosan nanospheres were preserved at 4°C for more than 3 months, and the image was taken by SEM as described above. The integrity of two types of nanospheres was then evaluated by analyzing SEM pictures captured at different stages.

### Release Characteristics *In Vitro*

As referenced to a previous study (44), the release characteristics of TgSDRO-pVAX1/PLGA and TgSDRO-pVAX1/PLGA nanospheres were conducted with minor modifications. Briefly, after being dissolved in 1 ml of PBS (pH 7.4), 2 mg nanospheres were dispersed on a rotary shaker (180 rpm) at 37°C. The time interval between two sample points was 12 h. To record the total volume of the supernatant and sample the supernatant, the nanospheres solution was centrifuged at 12,000 rpm for 5 min. The nanospheres that settled at the bottom were resuspended, and the tubes were put back. Referenced to the same PBS used for dissolving empty PLGA or chitosan nanospheres (loaded with PBS, instead of TgSDRO-pVAX1 plasmid), the concentrations of plasmid in the supernatant were immediately measured. Then, the cumulative release (CR) was calculated by formula (3). Each group had three replications, and each replication was measured once.

3 $$\text{CR}(\%)=\frac{\text{Total volume of supernatant}\times \text{Plasmid concentration}}{\text{Total weight of nanospheres}\times \text{LC}}\times 100\%$$

### Quantification of *T. gondii* in Tissue

Three BALB/c mice were challenged with 200 tachyzoites of *T. gondii* RH strain intraperitoneally. Seven days later, infected mice were sacrificed under the supervision of the Animal Ethics Committee, Nanjing Agriculture University, China. Then tissue samples from the intestine, heart, leg muscle, brain, liver, spleen, lung, and kidney were collected and store at -20°C until use.

To detect *T. gondii* in different tissues, PCR was conducted to amplify the internal transcribed spacer 1 (ITS-1) sequence based on a published paper (45). Briefly, 30 mg of tissue samples was used for genomic DNA extraction according to the instruction (OMEGA Bio-tek, Norcross, Georgia, USA), and the extracts were stored at -20°C until use. The PCR system was the same as that described in the section *Construction of the Eukaryotic Expression Plasmid*, and the amplification was determined by using the following program: preheating (5 min at 95°C), amplification for 40 cycles of 30 s at 95°C, 30 s at 55°C, and 30 s at 72°C, and the final extension (5 min at 72°C). The positive genomic DNA was obtained from the tachyzoites described in the section *Animals, Cell Lines, and Parasites* using the same method described above. In each amplification, both positive and negative controls (no-DNA control) were included. Then, 4 μl PCR amplicons were visualized in a 1.0% agarose gel containing 0.01% ethidium bromide.

To evaluate the parasitic load in tissues, absolute quantitative real-time PCR (qPCR) was conducted to illustrate the 529 bp repeat element in the extracts referenced to a published study (46)‘ absolute quantitative real-time PCR (qPCR) was conducted to illustrate the 529 bp repeat element in the extracts. The recombinant vector containing the amplified region was also constructed, and the copy numbers of cloned vectors were calculated by using an online tool (http://cels.uri.edu/gsc/cndna.html). For qPCR amplification, 10 μl of 2 × ChamQ SYBR qPCR MasterMix (Vazyme, Nanjing, China), 0.4 pmol of each primer, 0.4 μl 50×ROX Reference Dye 2, 1 μl of DNA extracts, and ddH_2_O to make a final volume of 20 µl were involved in each reaction. Each reaction was amplified by Applied Biosystems 7500 (Life Technologies, Carlsbad, USA) using the following program: pre-denaturation at 95°C for 30 s, followed by 40 cycles of 95°C for 10 s and 60°C for 30 s. A melting curve analysis was performed at the end of the reaction between 60°C and 95°C with an increment of 0.05°C/s. The melting curve of each amplification was ensured with one uniform peak at the expected temperature before further analysis. Before amplification, the OD260/OD280 value of each sample was analyzed by a nanodrop microvolume spectrophotometer. Each tissue had three replications, and each replication was performed once. Moreover, the recombinant vectors with a known copy number were also conducted in triplicate.

### Immunization and Challenging Schedules in Mice

BALB/c mice were randomly divided into five groups (28 mice/group), and all mice were immunized intramuscularly in the leg muscles with multipoint at 2-week intervals. For single immunization, the immunized dosage of each mouse did not exceed 0.2 ml. Mice were immunized with an equal volume of PBS that was used as the blank control (**Table 1**). To investigate the adverse reactions in animals, the living conditions of animals were observed weekly from the first immunization to challenge.

**Table 1:** Immunization formulations in mice.

Group	Treatment (each mouse)	Immunization	*T. gondii* infection
Blank	Equal volume of PBS	Intramuscular injection in the leg muscles at weeks 0 and 2	Intraperitoneal infections with 10^3^ tachyzoites for each mouse at week 4
Control	100 μg pVAX1 plasmid		
TgSDRO-pVAX1	100 μg TgSDRO-pVAX1 plasmid		
TgSDRO-pVAX1/CS	TgSDRO-pVAX1/CS nanospheres containing 100 μg TgSDRO-pVAX1 plasmid		
TgSDRO-pVAX1/PLGA	TgSDRO-pVAX1/PLGA nanospheres containing 100 μg TgSDRO-pVAX1 plasmid		

Two weeks after the last immunization, the immune protections were evaluated by a method described previously (47). In short, five mice in every group were challenged with a lethal dose (10^3^ tachyzoites) of *T. gondii* RH strain intraperitoneally. The infected mice in each group were sacrificed under supervision 7 days after the challenge, and the specific tissue was collected and stored at -20°C until use.

### Antibody and Cytokine Assays

Serum samples were collected from the eye socket at weeks 0, 2, and 4 before immunization and challenge. All serum samples were kept in -20°C before use. To obtain the soluble tachyzoite antigens (STAg), a previously described procedure was conducted (48).

According to a previous study (49), enzyme linked immunosorbent (ELISA) assays were performed to determine the *T. gondii*-specific serum antibody levels. Briefly, the 96-well plates (Costar, Cambridge, MA, USA) were coated with 1 μg STAg (diluted to 10 μg/ml with carbonate buffer pH 9.6) for each well overnight at 4°C. After being washed three times with TBST (TBS containing 0.05% Tween 20), each well was blocked with TBS containing 5% skimmed milk at 37°C for 1 h. Then mouse sera were diluted (1:100 in TBS) and added to each well for 1 h at 37°C after three times washing in TBST. After being subsequently rinsed 5 min in TBST, each well was incubated with HRP-conjugated anti-mouse IgG, IgG1, or IgG2a (1:5,000, eBioscience, San Diego, USA) at 37°C for 1 h to detect bound antibodies. Finally, 3,3’,5,5’-tetramethylbenzidine (TMB, Tiangen, Beijing, China) was used to evaluate the enzymatic activity; the reaction was stopped by adding 100 μl, 2 M newly prepared H_2_SO_4_. The absorbance was measured at 450 nm with a microplate photometer (Thermo Scientific, Waltham, USA). Each group had five replications, and each replication was measured once.

To assay the cytokine production levels in sera, commercially available ELISA kits (Senbeijia, Nanjing, China) were used strictly following the manufacturer’s instructions. Referenced to standard curves based on known amounts of mouse recombinant cytokines, the concentrations of interferon-gamma (IFN-γ) and interleukin (IL) 4 (IL-4), IL-10, and IL-17 were measured. The analysis was performed based on five replications in each group, and each replication was measured once.

### Determination of Lymphocyte Proliferation

One week after the booster immunization, three mice of each group (without challenge) were sacrificed under supervision to obtain the splenic lymphocytes by using a lymphocyte separation kit (Solarbio, Beijing, China). The splenic lymphocytes were adjusted to 10^5^ cells/well in the medium containing recombinant protein obtained in the section *Recombinant Plasmids Transfection in Vitro* and cultured in a 96-well plate. The plate was incubated for 72 h at 37°C. Then 10 μl of Cell Counting Kit 8 (CCK-8, Beyotime, Shanghai, China) was added according to the instructions. After 2-h incubation, the lymphocyte proliferation absorbance was measured at 450 nm with a microplate photometer. Each group had three replications, and each replication was measured once.

### Flow Cytometry Analysis

At weeks 0, 2, and 4, five mice from each group (before immunization and challenge) were euthanized to obtain the splenic as described in the section *Determination of Lymphocyte Proliferation*. The splenic lymphocytes were divided into several parts. For the maturation detection of dendritic cells (DCs), the splenic lymphocytes were cultured in DMEM containing 10% FBS and 1% double antibiotics for 12 h, and the non-adhering cells were discarded. After the plate was washed three times using PBS, the attached cells were collected by pipetting repeatedly and gently. Cells were then stained with anti-mouse CD11c-APC, CD86-FITC, and CD83-PE (eBioscience, San Diego, CA, USA). To investigate the MHC molecule changes in DCs, the splenic lymphocytes were stained with anti-mouse CD11c-PE, MHC-I-FITC, and MHC-II-APC (eBioscience, San Diego, CA, USA). To analyze the percentages of CD4^+^ T lymphocyte subsets, the splenic lymphocytes were stained with anti-mouse CD3e-PE and CD4-FITC (eBioscience, San Diego, CA, USA). To detect the percentages of CD8^+^ T lymphocyte subsets, the splenic lymphocytes were stained with anti-mouse CD3e-PE and CD8-FITC (eBioscience, San Diego, CA, USA). All stained procedures were conducted at 4°C for 40 min. After centrifugation, collection, and washing, cells were sorted by a flow cytometry (Beckman Coulter Inc, Brea, CA, USA). Each group had five replications, and each replication was measured once.

### Parasite Burdens in Mice

To determine the parasitic loads in mice, 30 mg of specific tissue obtained in the section *Immunization and Challenging Schedules in Mice* was used for genomic DNA extraction and qPCR as described in the section *Quantification of T. gondii in Tissue*. Each group had five replications, and each replication was performed in triplicate; the recombinant vectors with a known copy number were also conducted in triplicate.

### Data Analysis

Significance analysis was conducted by the SPSS 25.0 software (SPSS Inc., Chicago, IL, USA) and GraphPad 6.0 software (GraphPad Prism, San Diego, CA, USA). Based on one-way analysis of variance (ANOVA), differences between groups were considered as significant at *p* < 0.05. Based on the homogeneity of variance, values between the TgSDRO-pVAX1/CS and TgSDRO-pVAX1/PLGA groups were compared with the independent *t*-test. The flow cytometric analysis was illustrated by the CytExpert software (version 2.3, Beckman Coulter Inc, Brea, CA, USA).

## Results

### Recombinant Plasmids Construction and Expression

**Figure 1:**
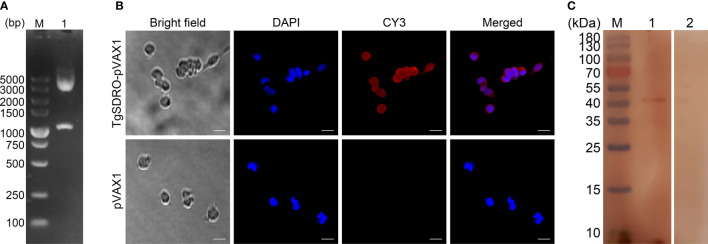
**(A)** Double digestion analysis of the recombinant plasmid TgSDRO-pVAX1. Line M: DL5000 marker; Line 1: Double digestion with *Eco*RI and *Xho*I. **(B)** Immunofluorescence analysis of the expression of TgSDRO-pVAX1 in HEK 293-T cells. Bar represented 25 μm. **(C)** Western blot was conducted to detect the expression of TgSDRO in HEK 293-T cells. Cell lysates of HEK 293-T cells transfected with TgSDRO-pVAX1 (Line 1) and pVAX1 (Line 2) detected by sera from *T. gondii*-infected rats. Line M: molecular weight (MW) marker proteins.

The recombinant plasmid TgSDRO-pVAX1 was successfully constructed as mentioned above. To verify the recombinant plasmid, an enzyme digestion was performed with *Eco*RI and *Xho*I, yielding two fragments of 1,119 and 2,966 bp (**Figure 1A**). The sequence analysis also proved that the insert in the recombinant plasmid was the ORF of TgSDRO. Together, all these results indicate that the recombinant plasmid was constructed correctly.

The endotoxin free DNA plasmids were purified by a commercial kit, and the endotoxin level of purified TgSDRO-pVAX1 plasmids fell to 0.1 EU/ml. Then, immunofluorescence assay was conducted to evaluate the *in vitro* expression of TgSDRO-pVAX1 at 72 h after transfection of HEK 293-T cells. Cells transfected with TgSDRO-pVAX1 showed specific red fluorescence, whereas cells transfected with pVAX1 did not display red fluorescence (**Figure 1B**). The western blotting results revealed a single band (approximately 41 kDa) of the expected molecular size (40.37 kDa), and no protein band was observed in cells transfected with the empty pVAX1 vector (**Figure 1C**). All these findings indicated that recombinant TgSDRO protein was expressed in HEK 293-T cells.

### Physical Characterization

**Figure 2:**
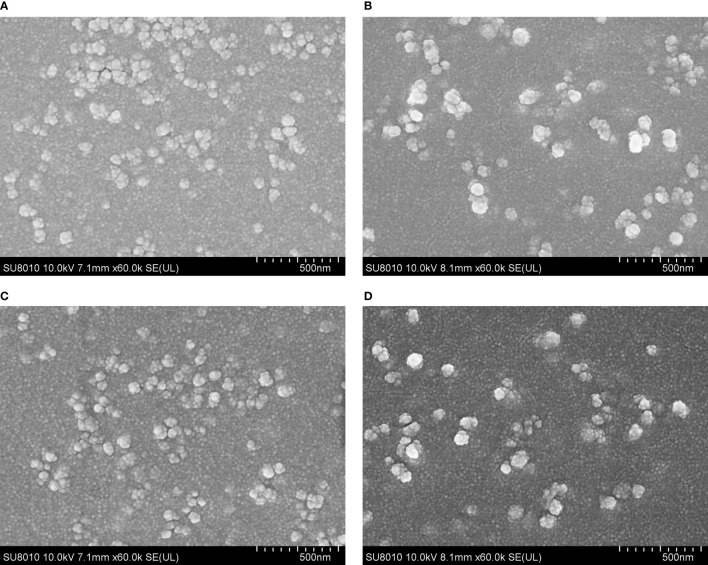
The morphology of TgSDRO-pVAX1/PLGA and TgSDRO-pVAX1/CS nanospheres. Freshly prepared TgSDRO-pVAX1/PLGA nanospheres **(A)** and those preserved over 3 months **(C)** were synthetized by a double emulsion solvent evaporation technique, while newly prepared TgSDRO-pVAX1/CS nanospheres **(B)** and those preserved over 3 months **(D)** were prepared by an ionic gelation technique (bar represented 500 nm).

The SEM images showed that both TgSDRO-pVAX1/PLGA and TgSDRO-pVAX1/CS nanospheres were spherical in shape with round convex particles in the surface (**Figure 2**). The mean diameter of TgSDRO-pVAX1/PLGA nanospheres formed using 6% PVA was about 100.96 ± 21.58 nm (n = 5), while the mean diameter of TgSDRO-pVAX1/CS nanospheres formed using 2 mg/ml TPP was about 115.77 ± 24.87 nm (n = 5). Based on five independent trails, the EE and LC of TgSDRO-pVAX1/PLGA nanosphere formulation reached 67.39% and 4.38% with the concentration of plasmids at 1 mg/ml, while those were 82.42% and 1.23% in TgSDRO-pVAX1/CS nanosphere formulations. Preserved at least 3 months (**Figures 2C, D**), TgSDRO-pVAX1/CS and TgSDRO-pVAX1/PLGA nanospheres were similar with those prepared freshly in morphologic characteristics. This finding suggested that both freeze-dried TgSDRO-pVAX1/CS and TgSDRO-pVAX1/PLGA nanospheres could keep the integrality over a long period of time at 4°C.

### Release Characteristics

**Figure 3:**
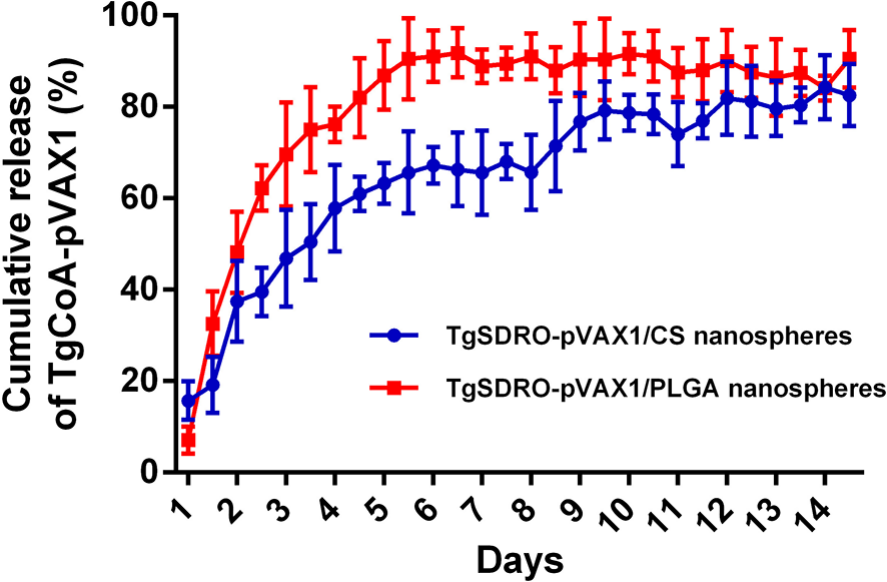
The release profile of TgSDRO-pVAX1/PLGA and TgSDRO-pVAX1/CS nanospheres *in vitro* over a 14-day period. Each sample was conducted once. Bar represents mean ± SD (n = 3).

By using a nanodrop microvolume spectrophotometer, the release characteristics of TgSDRO-pVAX1/PLGA and TgSDRO-pVAX1/CS nanospheres were further evaluated. TgSDRO-pVAX1/CS nanospheres showed a more favorable release profile compared to TgSDRO-pVAX1/PLGA nanospheres (**Figure 3**). Only 15.75 ± 4.19% of the antigens were released from TgSDRO-pVAX1/CS nanospheres immediately and slowly released over the following days, finally reaching 82.58 ± 6.79%. A quickly releasing phase of TgSDRO-pVAX1/PLGA nanospheres could be observed approximately during the first week with the initial CR at 7.09 ± 2.98%; then the release profile became flat and finally reached 90.50 ± 6.28% in the following days.

### Quantification of *T. gondii* in Tissues

**Figure 4:**
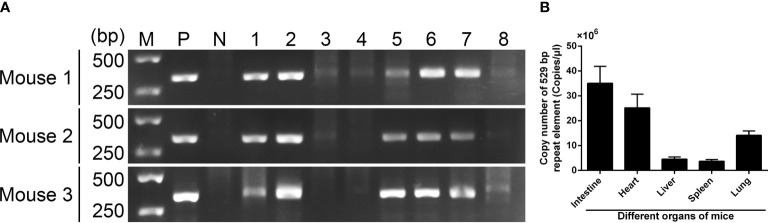
*T. gondii* detection in mouse tissues 7 days after infection. **(A)** *T. gondii* was detected by PCR based on ITS-1 sequence. Line M: DL5000 marker; Line P: positive control; Line N: negative control; Line 1-10: intestine, heart, leg muscle, brain, liver, spleen, lung, and kidney tissue. **(B)** *T. gondii* was detected by absolute qPCR based on a 529 bp repeat element. Results were showed as mean ± SD (n = 3).

To determine *T. gondii* infection in different tissues, PCR amplification was conducted based on the ITS-1 sequence. As described in **Figure 4A**, *T. gondii* was observed in the intestine, heart, liver, spleen, and lung, while the leg muscle, brain, and kidney tissue were not detected. To determine the amount of *T. gondii* in different tissues, absolute qPCR was conducted based on the 529 bp repeat element. Before qPCR amplification, the OD260/OD280 value of each sample was quantified, and all samples were in the range of 1.6–1.8. As illustrated in **Figure 4B**, the concentration of *T. gondii* reached a maximum in the intestine tissue (35.055 ×10^6^ copies/μl), followed by heart (25.179 ×10^6^ copies/μl), lung (14.085 ×10^6^ copies/μl), liver (4.529 ×10^6^ copies/μl), and spleen tissue (3.706 ×10^6^ copies/μl).

### Antibodies and Cytokine Production in Mice

**Figure 5:**
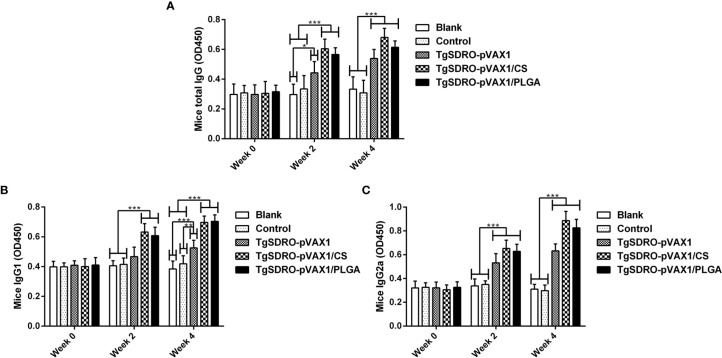
Immunoglobulin determination of total IgG **(A)**, isotypes IgG1 **(B)**, and IgG2 **(C)** in the sera from immunized mice at weeks 0, 2, and 4. Each sample was conducted once, and values were estimated using one-way ANOVA followed by Dunnett’s test and shown as the mean of the OD450 ± SD (n = 5). Values between the TgSDRO-pVAX1/CS and TgSDRO-pVAX1/PLGA groups were compared with the independent *t*-test. *p < 0.05, **p < 0.01, and ***p < 0.001 compared with the blank or control group.

The titers of total IgG, IgG1, and IgG2a induced in mice were measured by standard ELISA procedure. Demonstrated in **Figure 5A**, enhanced secretions of total IgG antibodies were obtained significantly in TgSDRO-pVAX1, TgSDRO-pVAX1/CS, and TgSDRO-pVAX1/PLGA after the first (week 2) and booster (week 4) immunization. Mice immunized with TgSDRO-pVAX1/CS could generate the highest titer of total IgG after the second immunization (week 4). Compared with the blank and control group, mice immunized with TgSDRO-pVAX1, TgSDRO-pVAX1/CS, and TgSDRO-pVAX1/PLGA could generate higher titers of isotype IgG1 (**Figure 5B**) and IgG2a (**Figure 5C**) after the first and second immunizations. Furthermore, no significant difference (*p* > 0.05) was evaluated in the TgSDRO-pVAX1/CS and TgSDRO-pVAX1/PLGA groups.

**Figure 6:**
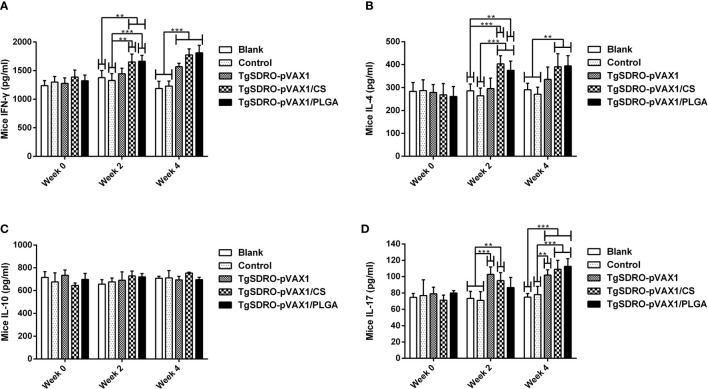
The dynamics of cytokine production in immunized mice. The mice sera were collected at weeks 0, 2, and 4, and the commercial ELISA kits were used to investigate the levels of IFN-γ **(A)**, IL-4 **(B)**, IL-10 **(C)**, and IL-17 **(D)** in mice sera. Each serum was investigated once. Results were estimated using one-way ANOVA tracked by Dunnett’s test, and values were shown as mean ± SD (n = 5). Based on the independent *t*-test, values between the TgSDRO-pVAX1/CS and TgSDRO-pVAX1/PLGA groups were compared. **p < 0.01 and ***p < 0.001 compared with the blank or control group.

Strictly following the instruction, the concentrations of IFN-γ, IL-4, IL-10, and IL-17 in the sera were determined by commercial ELISA kits based on the double antibody sandwich method. As demonstrated in **Figures 6A, B**, remarkable increases could be observed in the TgSDRO-pVAX1/CS and TgSDRO-pVAX1/PLGA groups after the first immunization (week 2), while the TgSDRO-pVAX1 group remained unchanged (*p* > 0.05). After the second immunization (week 4), all mice from the treatment group could generate a significant (*p* < 0.001) level of IFN-γ, and mice immunized with TgSDRO-pVAX1/CS and TgSDRO-pVAX1/PLGA could generate a significant (*p* < 0.01) level of IL-4. As for IL-17 showed in **Figure 6D**, significant secretions can be obtained in mice immunized with TgSDRO-pVAX1 and TgSDRO-pVAX1/CS after the first immunization, and after the second immunization, secretions of IL-17 were significantly enhanced in all treatment groups. Furthermore, equivalent IL-10 (*p* > 0.05, **Figure 6C**) secretions were observed in all immunized animals.

### Flow Cytometry Analysis in Dendritic Cells

**Figure 7:**
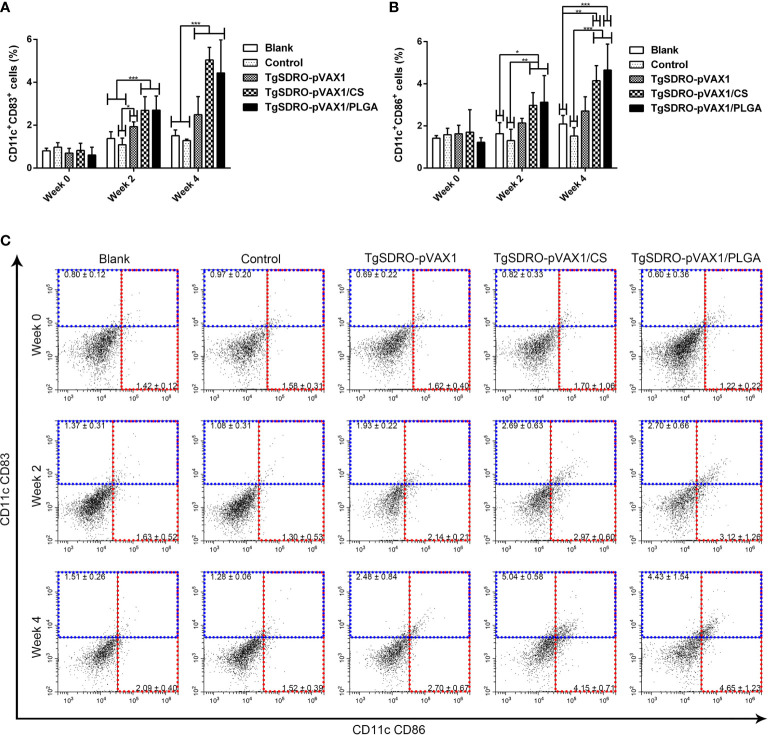
Analysis of DC maturation at weeks 0, 2, and 4 by flow cytometry. Five mice in each group were sacrificed; the bar graph showed the ratio of CD83 **(A)** and CD86 **(B)** molecules on splenic DCs, and the dot plots **(C)** showed the percentage of CD11c^+^ CD83^+^ and CD11c^+^ CD86^+^ cells. Each sample was conducted once. Results were estimated by one-way ANOVA followed by Dunnett’s test, and values were showed as mean ± SD (n = 5). Values between the TgSDRO-pVAX1/CS and TgSDRO-pVAX1/PLGA groups were compared with the independent *t*-test. *p < 0.05, **p < 0.01, and ***p < 0.001 compared with the blank group or the control group.

The expression of CD83 and CD86 on DCs was examined by flow cytometry. After the first and second immunizations (**Figures 7A, C**), treatment with TgSDRO-pVAX1, TgSDRO-pVAX1/CS, and TgSDRO-pVAX1/PLGA showed significant improvements in the percentage of CD11c^+^CD83^+^ cells when compared with the blank or control group. Furthermore, statistical improvements in the percentage of CD11c^+^CD86^+^ cells could be detected in the TgSDRO-pVAX1/CS and TgSDRO-pVAX1/PLGA groups (**Figures 7B, C**) after the first and second immunizations. Noticeably, no statistical difference (*p* > 0.05) was revealed between the TgSDRO-pVAX1/CS and TgSDRO-pVAX1/PLGA groups in DC maturation analysis.

**Figure 8:**
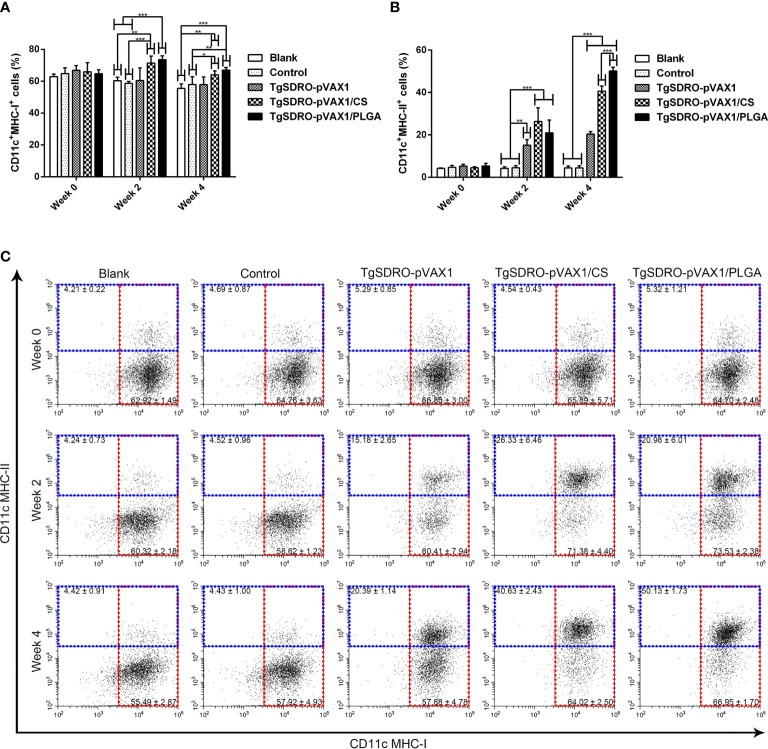
Analysis of MHC molecules at weeks 0, 2, and 4 by flow cytometry. Five mice in each group were sacrificed; the bar graph showed the ratio of MHC-I **(A)** and MHC-II **(B)** molecules on splenic DCs, and the dot plots **(C)** showed the percentage of CD11c^+^MHC-I^+^ and CD11c^+^MHC-II^+^ cells. Each sample was conducted once. Results were estimated by one-way ANOVA followed by Dunnett’s test, and values were showed as mean ± SD (n = 5). Values between the TgSDRO-pVAX1/CS and TgSDRO-pVAX1/PLGA groups were compared with the independent *t*-test. *p < 0.05, **p < 0.01, and ***p < 0.001 compared with the blank group or the control group.

In order to investigate the percentage of MHC molecules on splenic DCs, five mice from each group were sacrificed, and the spleen lymphocytes were analyzed. Described in **Figures 8A, C**, the ratio of MHC-I molecules in the TgSDRO-pVAX1/CS and TgSDRO-pVAX1/PLGA groups was enhanced after the first and second immunizations, while the TgSDRO-pVAX1 group remained unchanged (*p* > 0.05). For the ratio of MHC-II molecules in **Figures 8B, C**, significant enhancement was detected after the first and second immunizations. Furthermore, the statistics also revealed that mice immunized with TgSDRO-pVAX1/PLGA could generate significantly higher percentage (*p* < 0.001) of MHC-II molecules after a booster immunization compared to those immunized with TgSDRO-pVAX1/CS.

### Lymphocyte Proliferation

**Figure 9:**
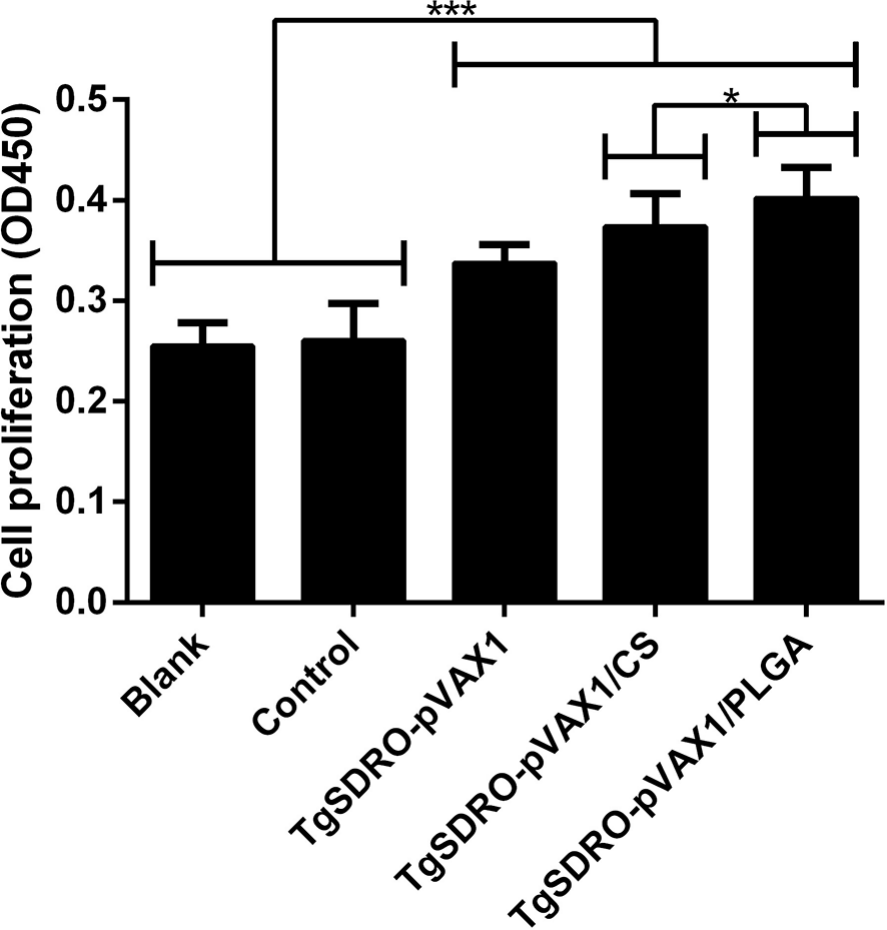
Splenocyte proliferation of different immunized mice. Three mice in each group were sacrificed, and spleen lymphocytes of each mouse were divided into four samples; the spleen lymphocytes were cultured using the medium from HEK 293-T lysate transfected with PBS (blank group), pVAX1 blank plasmid (control group), TgSDRO-pVAX1 recombinant plasmid (TgSDRO-pVAX1, TgSDRO-pVAX1/CS, and TgSDRO-pVAX1/PLGA groups). Each sample was measured once, and the bar graph was evaluated by one-way ANOVA followed by Dunnett’s test; values were showed as mean ± SD (n = 3). Values between the TgSDRO-pVAX1/CS and TgSDRO-pVAX1/PLGA groups were compared with the independent *t*-test. *p < 0.05 and ***p < 0.001 compared with the blank group or the control group.

Spleen lymphocytes were prepared from different groups 1 week after the second immunization, and the proliferative responses were analyzed. Evaluated in **Figure 9**, mice immunized with TgSDRO-pVAX1, TgSDRO-pVAX1/CS, and TgSDRO-pVAX1/PLGA produced a significant (*p* < 0.001) proliferation when compared with the blank and control group. Moreover, the TgSDRO-pVAX1/PLGA group could generate a higher (*p* < 0.05) proliferation than the TgSDRO-pVAX1/CS group.

### Flow Cytometry Analysis in T Lymphocytes

**Figure 10:**
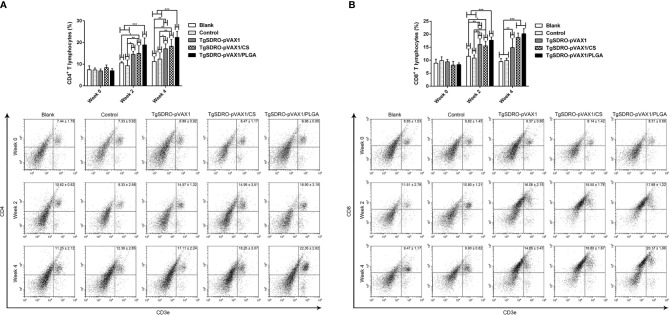
The proportions of CD4^+^ and CD8^+^ T lymphocytes in all groups. Five mice in each group were sacrificed, and spleen lymphocytes from each animal were stained with CD3e-PE, CD4-FITC **(A)** or CD3e-PE, CD8-FITC **(B)**. Determined by flow cytometry, each sample was conducted once. Results were estimated by one-way ANOVA followed by Dunnett’s test, and values were showed as mean ± SD (n = 5). Values between the TgSDRO-pVAX1/CS and TgSDRO-pVAX1/PLGA groups were compared with the independent *t*-test. *p < 0.05, **p < 0.01, and ***p < 0.001 compared with the blank group or the control group.

Five mice from each group were sacrificed and the splenocytes were stained, then the percentages of CD4^+^ T and CD8^+^ T lymphocytes of each group were conducted. Illustrated in **Figure 10A**, the percentage of CD4^+^ T cells in the TgSDRO-pVAX1, TgSDRO-pVAX1/CS, and TgSDRO-pVAX1/PLGA groups was significantly increased at weeks 2 and 4 when compared with the blank and control group. In the case of the percentage of CD8^+^ T cells (**Figure 10B**), the TgSDRO-pVAX1, TgSDRO-pVAX1/CS, and TgSDRO-pVAX1/PLGA groups generated a significant increase when compared with the blank and control group at weeks 2 and 4. For the percentages of CD4^+^ T and CD8^+^ T lymphocytes, mice in the TgSDRO-pVAX1/CS group could generate statistically similar (*p* > 0.05) to those in the TgSDRO-pVAX1/PLGA group after the first and second immunizations.

### Adverse Reaction and Parasite Burden in Animals

The animals’ health condition was investigated through the clinical observation, and the significant toxicity of nanospheres was not evaluated. All immunized animals were similar in physical health and stable in mental status over the duration of the trials compared with the animals immunized with PBS.

**Figure 11:**
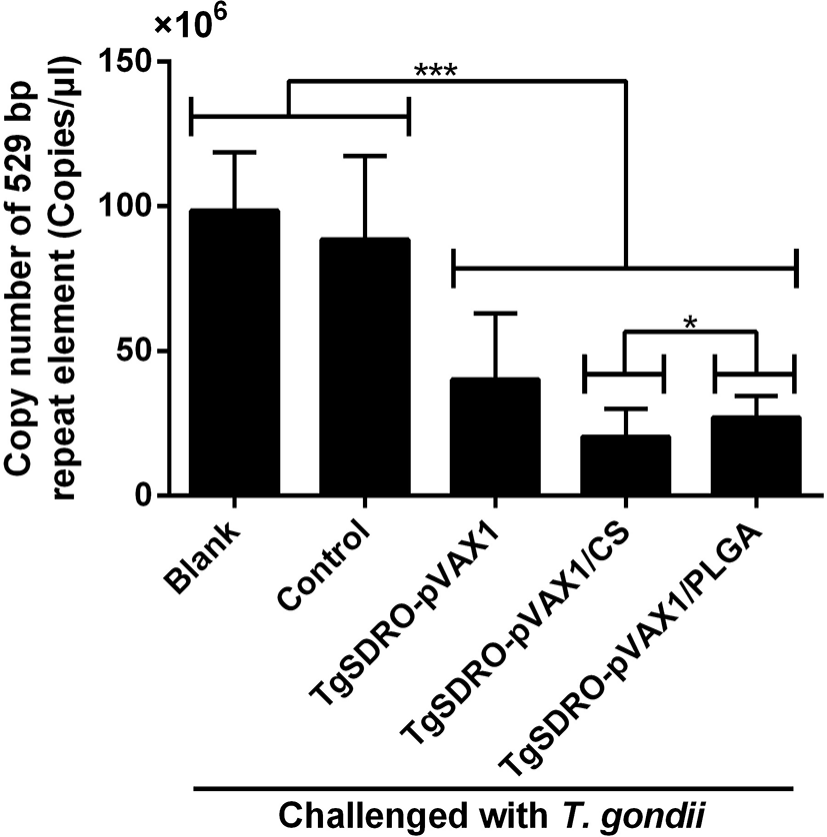
Copy number of a 529 bp repeat element in cardiac muscles from mice. Mice were intraperitoneally injected with 10^3^ tachyzoites of the highly virulent *T. gondii* RH strain at week 4 (2 weeks after the last immunization). Seven days after the challenge, five mice in each group were sacrificed, and cardiac muscles from the tip of the heart were collected. Each sample was performed three times. Results were evaluated using one-way ANOVA followed by Dunnett’s test, and the values were shown as mean ± SD (n = 5). Values between the TgSDRO-pVAX1/CS and TgSDRO-pVAX1/PLGA groups were compared with the independent *t*-test. *p < 0.05 and ***p < 0.001 compared with the blank group or the control group.

To obtain a more accurate analysis of parasite burden in mice, the method of absolute qPCR was conducted to detect the 529 bp repeat element of *T. gondii* in cardiac muscles from the tip of the heart. Before the qPCR amplification, the OD260/OD280 value of each sample was quantified, and all samples were in the range of 1.6–1.8. Presented in **Figure 11**, the parasite burden was significantly (*p* < 0.001) inhibited in the TgSDRO-pVAX1, TgSDRO-pVAX1/CS, and TgSDRO-pVAX1/PLGA groups compared to the blank and control group. Furthermore, significance (*p* < 0.05) also could be observed in the 529 bp repeat element between the TgSDRO-pVAX1/CS and TgSDRO-pVAX1/PLGA groups.

## Discussion

It has been proved that nanosphere delivery systems can prevent antigens from degradation and enable co-immunization of antigens and immunomodulators to the same cell type (50, 51). Encapsulated in PLGA nanospheres, co-delivery of antigens along with toll-like receptor (TLR) ligands can result in the secretion of natural killer (NK) cell-activating cytokines (52), and the bias toward Th1 and Th2 responses will be determined by the appropriate selection of TLR ligands (53). Unfortunately, the anionic nature of PLGA nanospheres can lead to their poor mucoadhesiveness (54), and the adjuvanticity can also be affected by their size, surface characteristics, or encapsulated antigens (55). By limiting nontarget immunity with the normal immune functions, chitosan nanospheres are considered as a powerful polymer candidate with high targeted immunogenicity (56, 57). Through the cyclic GMP-AMP synthase (cGAS) stimulator of interferon genes (STING) pathway, chitosan has been illustrated to be effective in activating DCs and Th1 immune response (58). However, exhibiting a disadvantage for antigen delivery, the deacetylation of chitosan nanospheres can modify its solubility, its degradation, its hydrophobicity, and even its electrostatic interaction (59).

At present, many methods have been developed for the formulation of polymeric nanospheres against *T. gondii* based on different types of polymeric nanospheres. Different methods for developing polymeric nanospheres can affect the EE and LC, and EE is an important value to assess the quality of nanospheres. Chitosan nanospheres loaded with DNA vaccine synthetized by Zheng et al. (60) showed 92.8% EE and 63.7% LC with an average diameter of 145.5 nm. Similar to the method used in this study, the EE of PLGA-NPs-pDNA nanospheres reached 57.50% with an average diameter of 149.60 nm (61). The modified double emulsion solvent evaporation technique and ionic gelation technique were applied in the synthesis of PLGA and chitosan nanospheres at the present study, and the EE of TgSDRO-pVAX1/CS and TgSDRO-pVAX1/PLGA nanospheres were 82.42% and 61.39%, respectively. However, the LC of TgSDRO-pVAX1/CS and TgSDRO-pVAX1/PLGA nanospheres were 1.23% and 4.38%, respectively. Such differences may be due to different methods, and further studies should be focused on the LC improvement of the DNA vaccine. Furthermore, synthetized nanospheres were spherical with many convex structures on the surface and had an average diameter of around 100.96 nm (TgSDRO-pVAX1/CS nanospheres) and 115.77 nm (TgSDRO-pVAX1/PLGA nanospheres). Effected on the efficiency of target genes in transfection and expression, the diameter of nanospheres loaded with DNA vaccines is an important indicator in quality assessment (62, 63). Evaluated by Caco-2 cell lines, 100-nm-sized PLGA nanospheres provided a better uptake, showing 2.5 times higher uptake than 1,000-nm-sized nanospheres (64). The TgSDRO-pVAX1/CS and TgSDRO-pVAX1/PLGA nanospheres prepared in the present study were approximately 100 nm, which could contribute to the entrance of DNA vaccine into cells. As already proved, the residual moisture content often affects the stability and integrity of nanospheres both physically and chemically during a long-term preservation (65). Hence, thorough freeze-drying is a promising approach to better stabilize the antigen against degradation. However, lyophilization can generate a series of drying and freezing stresses, which could denature antigens to various degrees (66). Thus, subsequent studies are required in nanosphere formulation to protect the stability of antigens during both drying and freezing processes. Furthermore, the repeatability in nanosphere synthesis is a nonnegligible factor in clinic application. In the present study, two types of nanospheres were synthesized multiple times, and relevant characteristics were conducted according to various batches of nanospheres. However, this preparation does not represent a reliable synthesis method, and further studies are required.

As delivery vehicles, effective nanospheres can provide protection of entrapped antigens against undesirable degradation during transportation to target cells (67). Through a slow hydrolysis process, PLGA nanospheres could release their antigens with a burst release making the antigens to be quickly released in a short time (34). In addition, negatively charged chitosan nanospheres could form polymer–plasmid complexes by adhering to the cell surface, leading to a continuous payload release (68). In the present study, a steadier release profile of TgSDRO-pVAX1/CS nanospheres was obtained in comparison with TgSDRO-pVAX1/PLGA nanospheres. A steady release curve does not mean an effective delivery system for drugs or vaccines; notably, efficient nanospheres are often limited by the toxicity and the difficulty in preparing them (20). During the preparation of TgSDRO-pVAX1/CS and TgSDRO-pVAX1/PLGA nanospheres in this study, DCM used in the synthesis of PLGA nanospheres has been thought to be toxic and hard to remove thoroughly by evaporation at room temperature (69). To avoid toxicity, TgSDRO-pVAX1/PLGA nanospheres were completely lyophilized. As expected, TgSDRO-pVAX1/CS and TgSDRO-pVAX1/PLGA nanospheres did not show adverse reactions based on the clinical observations.

In a previous paper, the predilection tissue of *T. gondii* has been previously described in the heart of pigs (70), and another paper revealed that the lung and heart were detected to have a high parasite burden in pig models of *T. gondii* infection (71). However, the animals used to illustrate these results were infected orally, and no published paper reveals the predilection tissue of *T. gondii* in mice through intraperitoneal challenges. Based on the results in the present study, the intestine, heart, and lung tissue were detected to have a higher parasite burden compared to the liver and spleen tissue. As it had the highest parasitic loads infected with oocysts in previous studies (70, 72), the heart tissue was negative in PCR amplifications; such results may be due to different infection ways. Although the intestine tissue owned the highest parasite burden among all investigated tissues, the heart muscles were selected for the subsequent trials mainly due to the potential contamination of challenged *T. gondii* in the intestine tissue.

Associated with inhibiting cell absorption and promoting macrophage killing of *T. gondii*, it has been long recognized that humoral response plays a critical role in immune protection against *T. gondii* infection (73). In the present study, immunized mice could gradually generate high levels of total IgG, IgG1, and IgG2a. According to a published paper, high levels of total IgG are critical in anti-*T. gondii* infections and depressing cyst reactivation (74). In addition, the subclass IgG1 is associated with Th2-related immunity, and Th1-biased response is associated with subclass IgG2a (47, 75). Our results also indicated that two types of nanospheres could generate Th1-biased immune response with the IgG2a/IgG1 value higher than one after a boost immunization. Similar to our results, Th1-biased immune response was also illustrated in BALB/c mice vaccinated with pEGFP-N1-HBsAg-GRA1-ROP2 plasmids (17). Playing an important role in resisting intracellular infection, Th1-biased immunity has been proved essential in anti-*T. gondii* infections (76, 77).

Cytokines are critical in mediating the T helper (Th) cells, and enhanced secretion of Th1 cytokines plays an essential role in resisting *T. gondii* (78). Increased secretion of IFN-γ can depress the replications of *T. gondii* through activating phagocytes (79, 80). Compared with the blank and control group, animals from the vaccinated group were observed to have Th1 biased immunity by generating a higher level of IFN-γ after the second immunization. According to a published paper, the early mortality of acute toxoplasmosis is mainly induced by severe inflammation rather than *T. gondii* (81). Thus, cytokines IL-4 and IL-10 were conducted. As the factor of Th2 biased immunity, IL-4 could induce the secretion of IgG1 (82). A significant enhancement of cytokine IL-4 was observed in immunizations with two types of nanospheres. However, the level of IL-10 remained similar in all vaccinated groups. Together with the results of antibodies, these results demonstrated that TgSDRO-pVAX1/CS and TgSDRO-pVAX1/PLGA nanospheres could elicit both Th1 and Th2 immune responses and mainly skewed to a Th1 biased immunity, which played a critical role in cell-mediated immunity and *T. gondii* resistance (83, 84). In addition, as an inflammatory modulator produced by Th17 (85), cytokine IL-17 is also important in anti-*T. gondii* infections (86, 87). In the present study, animals from the vaccinated group were observed to have significantly higher levels of IL-17 after a booster immunization, indicating that TgSDRO-pVAX1 and its nanospheres were capable of triggering Th17 differentiation and involved in anti-*T. gondii* infections.

Being capable of antigen capturing and processing, DCs can efficiently activate naïve T cells against antigens and polarize them to different effector fates (88, 89). Immature DCs are poor activators and ineffective in T lymphocyte-stimulating activity, while the activity boosts hugely when DCs receive a maturation signal (90). When receiving a maturation signal, DCs experience massive changes, mainly including enhanced surface molecule expression critical for T lymphocyte activation, such as MHC-II, CD40, and CD86 (91). Furthermore, as a typical surface marker, CD83 was expressed in mature DCs and other cells and used as an immune checkpoint (92). In the present study, we obtained the differences in the relative percentages of CD11c^+^CD83^+^ and CD11c^+^CD86^+^ cells among the vaccinated, blank, and control groups. These findings indicated that TgSDRO-pVAX1/CS and TgSDRO-pVAX1/PLGA nanospheres could accelerate the maturation of DCs and launch their functions.

As the most effective antigen-presenting cells (APCs), DCs could evoke the innate and adaptive immune response and are critical in the interaction between two types of immunity (93, 94). To perform antigen-presenting functions, DCs have evolved MHC-II proteins, which could load processed antigens (95). Then antigen–MHC-II complexes are transported to the surface of cells, where they can be recognized by CD4^+^ T lymphocytes. Evidence from previous studies suggested that MHC-II could be critical in eliciting strong immune response (96, 97). In the present study, significantly higher percentages of CD11c^+^MHC-I^+^ and CD11c^+^MHC-II^+^ cells were evaluated in two types of nanospheres compared with the blank or control group. As a critical molecule in endogenous antigen-presenting cells, MHC-I could induce strong CD8^+^ T lymphocyte immunity (98). Furthermore, the promoted expression of MHC-I might be due to the high level of IFN-γ. Previous studies suggested that IFN-γ could enhance the MHC-I expression and antigen presentation by activating the Janus kinase/Signal transducer and activator of transcription 1 (JAK/STAT1) pathway (99, 100). To sum up, these results suggested that TgSDRO-pVAX1/CS and TgSDRO-pVAX1/PLGA nanospheres could activate the MHC-II restricted and MHC-I restricted antigen presentation.

The lymphocyte proliferation was regarded as the most efficient index in reflecting the status of immunity *in vivo* (101). In the present study, we observed remarkable differences in lymphocyte proliferation between the vaccinated, blank, and control groups. Furthermore, the proliferation ability from animals immunized with TgSDRO-pVAX1/PLGA nanospheres was significantly enhanced compared with animals immunized with TgSDRO-pVAX1/CS nanospheres. These findings indicated that both TgSDRO-pVAX1/CS and TgSDRO-pVAX1/PLGA nanospheres could effectively promote the proliferation of lymphocytes, and the PLGA delivery vehicle was more efficient.

Due to the intracellular inhibition of *T. gondii*, T lymphocyte activation is critical in resisting the replications of *T. gondii*, especially the CD4^+^ and CD8^+^ T lymphocytes (102). Previous studies have revealed that CD4^+^ T lymphocytes are essential in mediating immunity against toxoplasmosis, while CD8^+^ T lymphocytes play an important role in the cytotoxic effect (103, 104). In addition, CD4^+^ T lymphocytes play an essential role in formulations of memory CD8^+^ T lymphocytes after immunizations or infections (105). In the present study, the percentages of CD3e^+^CD4^+^ and CD3e^+^CD8^+^ cells were significantly promoted in all vaccinated mice compared to those in the blank and control group. This observation indicated that animals immunized with TgSDRO-pVAX1/CS and TgSDRO-pVAX1/PLGA nanospheres could elicit higher levels of CD4^+^ and CD8^+^ T lymphocytes, which may contribute to the humoral and cellular immunity against acute toxoplasmosis. The ability of two types of nanospheres to enhance the proportions of CD4^+^ and CD8^+^ T lymphocytes was similar to that noted in the previous studies (17, 31, 106).

The parasite burden of vaccinated animals against *T. gondii* challenge is regarded as the most direct indicator to assess a vaccine. Therefore, an acute infection model was established to analyze the *T. gondii* burden in cardiac muscles. In comparison with the blank and control group, animals from TgSDRO-pVAX1, TgSDRO-pVAX1/CS, and TgSDRO-pVAX1/PLGA groups presented an obvious inhibition in *T. gondii* infections, demonstrating the immune protection, which was similar to previous studies employing different DNA vaccines (17, 107, 108). In addition, our results also indicated that TgSDRO-pVAX1/PLGA nanospheres were more efficient than TgSDRO-pVAX1/CS nanospheres in resisting the replications of *T. gondii*. Thus, TgSDRO-pVAX1/PLGA nanospheres may have more potential for the prevention of acute toxoplasmosis.

## Conclusion

In summary, this study suggests that immunizations with TgSDRO-pVAX1/CS and TgSDRO-pVAX1/PLGA nanospheres could generate immune protection against acute *T. gondii* infection with a significantly lower parasite burden in animals. The regulated antibodies and cytokine secretions and the flow cytometry results suggest that TgSDRO-pVAX1/CS and TgSDRO-pVAX1/PLGA nanospheres could trigger Th1/Th2 cellular and humoral immunity in mice. As the potent delivery system, chitosan and PLGA can enhance cellular immunity by inducing Th1 biased immune responses. Furthermore, as the promising DNA vaccines, TgSDRO-pVAX1/CS and TgSDRO-pVAX1/PLGA nanospheres can generate similar immune protection and are substitutes for each other. However, the immune mechanisms of TgSDRO-pVAX1/CS and TgSDRO-pVAX1/PLGA nanospheres are complicated and required more studies. The subsequent studies on this promising DNA vaccine should improve the loading capacity to enhance the immune protection and shed light on its applicable conditions to other hosts and its protectivity against chronic toxoplasmosis.

## Data Availability Statement

The original contributions presented in the study are included in the article/supplementary material. Further inquiries can be directed to the corresponding author.

## Ethics Statement

The animal study was reviewed and approved by the animal ethics committee of the responsible authority from the College of Veterinary Medicine, Nanjing Agricultural University, China.

## Author Contributions

ZY: Investigation, data curation, writing—original draft, formal analysis, and methodology. WC: Data curation, investigation, and formal analysis. XG: Investigation, methodology, and writing—review and editing. MA: Data curation, investigation, and writing—review and editing. JLi: Data curation, methodology, and funding acquisition. JLu: Methodology and funding acquisition. RY: Methodology, supervision, and project administration. LX: Methodology, funding acquisition, and project administration. XS: Methodology, project administration, and writing—review and editing. XL: Conceptualization, data curation, funding acquisition, methodology, supervision, writing—original draft, and writing—review and editing. All authors contributed to the article and approved the submitted version.

## Funding

This research was supported by Key Scientific and Technological Project of XPCC (2020AB025), College Students’ innovation and entrepreneurship training program (202010307099), and State Key Laboratory of Veterinary Etiological Biology, Lanzhou Veterinary Research Institute, Chinese Academy of Agricultural Sciences.

## Conflict of Interest

The authors declare that the research was conducted in the absence of any commercial or financial relationships that could be construed as a potential conflict of interest.

## Publisher’s Note

All claims expressed in this article are solely those of the authors and do not necessarily represent those of their affiliated organizations, or those of the publisher, the editors and the reviewers. Any product that may be evaluated in this article, or claim that may be made by its manufacturer, is not guaranteed or endorsed by the publisher.
